# Expectations of clients, insurers, and providers: a qualitative responsiveness assessment among private health insurance sector in Kampala-Uganda

**DOI:** 10.1186/s12913-023-10386-x

**Published:** 2023-12-05

**Authors:** Tonny Tindyebwa, Richard Ssempala, Aloysius Ssennyonjo, Chrispus Mayora, Micheal Muhoozi, Joan Tusabe, Paul Mukama, Ssengooba Freddie

**Affiliations:** 1 Department of Disease Control and Environmental Health, School of Public Health, Makerere University, Kampala, Uganda; 2 Department of Economic Theory and Analysis, School of Economics, Makerere University, Kampala, Uganda; 3 Department of Health Policy Planning and Management, School of Public Health, Makerere University, Kampala, Uganda; 4 Department of Epidemiology and Biostatistics, School of Public Health, Makerere University, Kampala, Uganda

**Keywords:** Expectations, Responsiveness, Private health insurance, Client, Insurer, Provider

## Abstract

**Background:**

There is less attention to assessing how health services meet the expectations of private health insurance (PHI) actors, clients, insurers, and providers in developing countries. Interdependently, the expectations of each actor are stipulated during contract negotiations (duties, obligations, and privileges) in a PHI arrangement. Complementary service roles performed by each actor significantly contribute to achieving their expectations. This study assessed the role of PHI in meeting the expectations of clients, insurers, and providers in Kampala. Lessons from this study may inform possible reviews and improvements in Uganda’s proposed National Health Insurance Scheme (NHIS) to ensure NHIS service responsiveness.

**Methods:**

This study employed a qualitative case-study design. Eight (8) focus group discussions (FGDs) with insured clients and nine (9) key informant interviews (KIIs) with insurer and provider liaison officers between October 2020 and February 2021 were conducted. Participants were purposively selected from eligible institutions. Thematic analysis was employed, and findings were presented using themes with corresponding anonymized narratives and quotes.

**Results:**

Client-Provider, Client-Insurer, and Provider-Insurer expectations were generally not met. Client-provider expectations: Although most facilities were clean with a conducive care environment, clients experienced low service care responsiveness characterized by long waiting times. Both clients and providers received inadequate feedback about services they received and delivered respectively, in addition to prompt care being received by a few clients. For client-insurer expectations, under unclear service packages, clients received low-quality medicines. Lastly, for provider-insurer expectations, delayed payments, selective periodic assessments, and inadequate orientation of clients on insurance plans were most reported. Weak coordination between the client-provider and insurer did not support delivery processes for responsive service.

**Conclusion:**

Health care service responsiveness was generally low. There is a need to commit resources to support the setting up of clearer service package orientation programs, and efficient monitoring and feedback platforms. Uganda’s proposed National Health Insurance Act may use these findings to: Inform its design initiatives focusing on operating under realistic expectations, investment in quality improvement systems and coordination, and efficient and accountable client care relationships.

## Introduction

Health care service delivery models work toward meeting the expectations of the actors involved [1–3]. Health insurance, as a financial risk protection initiative, plays an important role in supporting the provision of responsive health services, especially in urban communities [4–6]. The list of service items and their limits purchased by the client-policy holder inform service procurement by the insurer from the provider. Such information informs the expectations 1 from each actor [7]. Consumption of such health care services is thereafter made reference to as stipulated in the client’s insurance policy [8]. If such a policy is executed as expected, each actor registers satisfaction since each of them will have executed their complementary roles interdependently well [3]. Eventually, service provision in a client-oriented manner is achieved, thus achieving service responsiveness [9]. Responsiveness in this study therefore refers to how actual experiences of the clients, Insurers and Providers aligned or misaligned to their initial expectations [3, 10].

During health system performance assessments, the World Health Organization (WHO) recommended the inclusion of expectation domains such as the soundness of facilities, prompt attention, communication, and dignified treatment [10]. Assessing such domains in a private health insurance (PHI) arrangement has received less attention in developing countries than in developed countries [11, 12]. A few client expectations with the exclusion of providers and insurers have been documented [13] Reports from countries such as Ghana and South Africa indicate low levels of service level responsiveness [14–16].

Less than 1% (0.8%) of Uganda’s population access private health insurance (PHI) services. Ten [10] licensed insurance organizations, [8] insurance companies and [2] health membership organizations (HMOs) offer these services through providers such as hospitals, clinics, pharmacies and medical laboratories [17]. In Kampala, service responsiveness under PHI is less known. Whereas 7% of city employers have supported approximately 4% of their employees with PHI covers, remedying their catastrophic expenditure on health [18], very little is known about how their expectations have been met. A National Household Survey report indicated the unavailability of medicines, long waiting time, a limited range of services, understaffing, inconvenient opening hours, staff absenteeism and consideration of the client’s culture or religion as key concerns raised by the population. These factors affect service responsiveness [19].

Negative attitudes of health workers and long waiting times while accessing care were also indicated in a study involving noninsured clients [20]. Responsive service is preferred in a PHI arrangement [21]. This study therefore aimed to explore the factors that shape the relationships and how the respective expectations among actors in the PHI were met in Kampala. The findings may provide a basis for reflection on the improved design of the proposed National Health Insurance Scheme (NHIS) and later its implementation while ensuring service responsiveness.

## Methods

### Study setting and sampling procedure

A qualitative case-study design was employed. We aimed to understand why unmet expectations under private health insurance existed [22, 23] among PHI actors in Kampala City.

Kampala was purposively selected because it is the capital city. It hosts many health care providers, insurance companies and a large urban workforce with access to health insurance cover [18, 24–26]. We identified the leading five health insurance organizations (by client volume) with guidance from the Uganda Insurance Regulatory Authority (IRA). We identified employing institutions that had running contracts with these insurance organizations. These were contacted through an authorization request for enrollment of potential respondents (employees) into the study.

Using staff communication platforms such as Noticeboards, emails and WhatsApp groups, a call for employees (clients) who had received heath care services from a Kampala-based provider network using their insurance cover in the last 12 months was shared, and those willing to participate were identified. The research team contact persons (administrators and human resource officers) compiled and shared their contact information with the research team. We contacted eligible participants both through phone calls and physically at their workstations and scheduled interview appointments. Employees (clients) whose insurance membership was not active in the past 12 months were not enrolled in the study.

Purposively identified service providers, a mix of private for profit (PFP) and private not for profit (PNFP) with assistance from employing institutions, were represented by their respective liaison persons (human resource officers for employing institutions, hospital administrators at provider facilities and medical insurance officers at insurer organizations). We were unable to identify employment institutions under Sanlam insurance organization. Some had closed premises due to COVID-19, while others did not give feedback to our study participation request. These details are summarized in Table 1.

**Table 1:** Illustration of the sampling process

Nominated Insurers	Identified Institution	Enrolled Institution	Enrolled clients	FGDs	HCPs	KIs
UAP Old Mutual	15	3	58	3	1	3
Jubilee Insurance	10	2	32	2	1	3
Prudential Assurance	8	1	10	1	1	2
Sanlam Life	6	0	0	0	1	0
AAR Medical	6	3	23	2	1	3
**Total**	**45**	**9**	**123**	**8**	**5**	**11**

At least two [2] focal point persons from both the employing institution and the health care provider were enrolled as key informants. These were presumed to be knowledgeable and experienced in addressing the study subject.

### Data collection

Eight (8) focus group discussions (FGDs) between 6 and 9 insured clients and nine (9) key informant interviews (KIIs) with liaison officers were held from October 2020-March 2021. We ensured heterogeneity among FGD participants by employment role. The developed semi-structured interview guides with predetermined open-ended questions that aligned to the themes under study [10, 27, 28] were used. Both the FGD and KII guides are composed of modified domains that are a measure of responsiveness [10]. These included waiting time, prompt attention, service eligibility, periodic assessments, timeliness of payments, payment mechanisms, communication, and soundness of facilities. Conducive offices, boardrooms and other welfare spaces were used for interviews. Prior to the FGDs, we obtained informed written consent from each participant followed by their sociodemographic characteristics. Number tags were given to each FGD participant for easy identification during the discussions. We used the FGD guide to elicit each participant’s understanding of their general health care service journey expectations. Thereafter, the tool guided the discussion on specific thematic service expectations of their providers and insurers. Key informant interviews also elicited information on actor-specific relationship expectations (Insurer of provider, Insurer of client, provider of insurer and provider of client) during health care service delivery under a private health insurance arrangement. In all the interviews, we probed participant responses for enriched in-depth clarifications. The contextual consideration of the interpretation of thematic domains during FGD was premised on the role theory perspective on dyadic interactions [29] of the actors within a health system [3]. Their duties, obligations and privileges based on individual actor positions were interdependent of those of another performing a complementary role [29]. Saturation was realized when the research team realized that no new information was being generated from the participants [27, 30]. We complied with both the Uganda National Council for Science and Technology (UNCST) 2020 guidelines on conducting research and the Uganda Ministry of Health Standard Operating Procedures (SOPs) in the context of COVID-19 [31, 32]. Physical distancing, proper face masking and use of alcohol-based hand sanitizers were ensured in preventing COVID-19 infection during all data collection exercises.

### Data management and analysis

We transcribed verbatim all audio tapped recording followed by proof reading of transcript to ensure consistency. Subsequently, they were exported to a computer-assisted data management software, ATLAS. ti version 6.0 for easy sorting and organizing the data [33]. An initial codebook was developed using a sample of transcripts by two experienced and trained individuals in health insurance and qualitative data management. The developed codebook was later applied to the entire atlas project. Newly emerging codes were discussed and resolved, and a decision to add them to the project was made during briefing meetings. To establish code pattern similarities among insurance actor relationship expectations and the magnitude of response categories, we extracted and utilized query reports and code-document tables. Thematic analysis was employed, and findings were presented using themes with corresponding anonymized narratives and quotes.

### Validity and reliability

To obtain a comprehensive situation of the expectations across the health insurance service delivery model, we interviewed all three actors: the client, the provider and the insurer. This enabled the research team to validate and triangulate results across the three actor relationships. Refresher training on the study of key health insurance concepts and qualitative data collection was provided to the study team prior to data collection with the aim of ensuring the collection of quality data.

We retrained the research team on good interviewing practices, such as keeping the natural flow of the discussions in a lively, gentle, and friendly manner. In scenarios where some individuals expressed disagreements during FGDs, the moderator re-emphasized the correctness of all submissions from each participant since they were based on their experiences.

For adequate interpretation of the findings, the research team was retrained on the use of reflexive journals to document both verbal and nonverbal responses. Debrief meetings after the day’s interviews were conducted, and structured summary reports were compiled to depict emerging areas of discussion from each interview. This reinforced the interviews to collect rich and diverse data. A pilot study among a sample of participants (2 FGD-insured clients, 3 KIIs; 2 health providers and 1 insurers) to evaluate study questions for their content appropriateness to the target population was conducted. Identified new codes and probes were based on participant responses. This helped in expanding the sample size to capture and analyze data from a diversity of backgrounds.

## Results

Table 2 presents the sociodemographic characteristics of the study participants in Kampala. Sixty-two (62) participants were enrolled in the study. Of these, 42 (68%) were females. The median age of the participants was 30 years. All participants had attained advanced training, with the diploma level being reached by the majority (25, 40%). Health workers 21 (34%) were the most interviewed, as shown in Table 2.

**Table 2:** Participant characteristics of FGDs and KIIs

Socio-demographic characteristic	Number
**Sex**	
Males	20
Females	42
**Age (Median = 30)**	
21–30	29
31–40	20
41–50	8
Above 51	5
**Education status**	
Diploma level training	25
Undergraduate training	19
Post Graduate training	18
**Participant employment roles**	
Health workers	21
Finance & Administration	29
Procurement & Logistics	12
**Participant category**	
Insurance organizations (KI’s)	4
Health Care Providers (KI’s: -3HMOs^a^, 2 non-HMOs)	5
Insured clients (FGDs)	53

^a^HMO refers to Health Management Organization. Health Management Organization (HMO) refers to a health insurance plan where care is provided through a network of health care providers that treat clients for a prepaid cost [34]

The expectations of health care services across actors as expressed by the client, insurer, and provider under the health insurance arrangement were interrelated. Table 3. Detailed findings on bilateral expectations from each set of actor-based dyadic interactions; the client-insurer, client-provider, and provider-insurance relationships are summarized under three research sections: (1) Expectations within the client-insurer relationship. (2) Expectations within the client-insurer relationship and (3) Expectations within the insurer-provider relationship. Table 3 shows a summary of findings across the three actors.

**Table 3:** Summary of key findings across the three actor relationships

Actor	Insurers	Providers	Clients
**Clients**	“Shallow policy orientation”	“Inadequate feedback”	**Not applicable**
	“Delayed facility payments”	“Prefer cash payments”	
	“Unclear benefit packages”^a^	“Long waiting time”	
		“Prompt care received by a few”	
		“Low quality medicines given”	
		“Clean Facilities”	
**Insurers**	**Not applicable**	“Compromised service delivery”	“Non-use of feedback platforms”
		“Little know of Insurance model”	“Received inception orientation”
		“Influence by cash economy”	“Explain policy less to their beneficiaries”
		“No service guides”	
**Providers**	“Selective periodic assessments”	**Not applicable**	“Poor attitude to correction”
	“Clear payment timelines”		“Un realistic expectations”
	“Less orientation on policy”		“Unethical suggestions to provider staff”

^a^Unclear benefit packages referred to service item lists and their limits

### Expectations within the client-insurer relationship

Respondents were critical on issues of service eligibility, payment timeliness and feedback mechanisms. Clients received inadequate orientation on their insurance policies, resulting in seeking care under inadequate breakdown of actual services and their limits (unclear benefit packages). Delayed payments to the provider facility by the insurer were also reported by clients.

#### Service eligibility - “shallow policy orientation” and “unclear benefit packages”

Insurers should orient clients before signing up for their specific service packages. The type, volume, and mode of delivery inform the clients’ expectations from the provider. In this study, clients sought care under unclear service volumes and limits. One of the clients whose knowledge on his insurance policy and service package and limit was unclear was quoted as saying,

…they say, insurance? Ohh!! Insurance is covering this much, for you, you will pay this much. So probably, there are certain things that the insurance does not cover and that was not made very clear. FGD 3 participant

#### “Received inception orientation” and “explain policy less to their beneficiaries”

In instances where some insurers oriented principal policy holders on their service volumes and limits, they expected the principal policy holders to extend the same briefing to their beneficiaries. Unfortunately, this seemed not to have been done; thus, the clients complained of having received a shallow policy orientation.

#### Timeliness of payment: “delayed facility payments”

The payment function is fulfilled by insurers in compensating providers for the volume of services that clients (patients) consume. Effecting timely payments in line with initial contract negotiations supports uninterrupted service delivery to meet client expectations. Clients reported delayed provider payments, which influenced the provider’s motivation to provide services to insured clients. They further mentioned that providers were instead prioritizing attending to cash patients. Some clients complained about nonpayment for the hospital by the insurers:

…but also, the case of nonpayment…, these insurance companies do not pay the hospitals, so those hospitals are not motivated enough to work on clients. …some get blunt and tell you that those ones (their insurer) don’t pay, so you ***kweyiya*** (find alternative ways to pay for your care) somehow. FGD 1 Participant

Another participant commented on the provider preference to cash patients instead of insured clients saying:

If the insurance doesn’t pay promptly, I don’t know what to use. Then, the facility is also happy in receiving cash. There are some people coming with cash, but for you, with your promises, they will not give you the same attention. They will see you as a burden. KII Participant

#### Feedback mechanisms- “inadequate feedback”

Providing feedback to insured clients on service delivery and its effective use is key to improving service responsiveness. Since insurers negotiate service provision on behalf of clients with the provider, their initiative to provide periodic feedback is very important. As an accountability mechanism, it builds confidence in the service delivery processes. In this study, the insurer’s inability to provide adequate feedback to insured clients on service consumption was strongly expressed. Some clients incurred unexpected expenses in purchasing services from the provider and expressed concerns about the untimely feedback.

…no one warns me that you are exceeding your (service consumption) limits…you pay more money from your salary. …feedback is that it is not timely; hence, it’s not helpful. FGD 3-Health workers

Another participant said that,

…they also wait on someone to give feedback, which takes very long. FGD 5-Participant

#### “Nonuse of feedback platforms”

On the other hand, insurers expected clients to proactively use feedback sharing platforms such as emails and contacts to register their concerns. However, this was not commonly done. Insures reported that clients were negligent in the use of some of the provided feedback platforms.

### Expectations within the client-provider relationship

The waiting time, prompt attention and soundness of facilities under this section were mostly reported. Whereas clients were very pleased with clean care environments at most provider facilities, they also expressed dissatisfaction with long waiting times. On the other hand, a few clients observed respectful care and attention (prompt care) from the provider.

#### Waiting time- “long waiting time”

The mode of service delivery at service points necessitates that clients are attended to at intervals. Client volumes and procedural processes influence the time between arrival and receipt of services. Proximate waiting time is considered a good indicator of quality services. In this study, the average waiting time was four to five hours (4–5 h). Clients reported high outpatient patient volumes in facilities as a major cause of long waiting time due to congestion and long approval processes. Additionally, clients with emergency conditions opted for cash payments to avoid delays. In some circumstances, socially well-known “connected” clients were observed to receive preferential care. They were attended to faster than the rest. Some clients commented about the high number of patients saying,

… there was congestion, because all kinds of patients come in from all different kinds of insurers. Therefore, the waiting time was longer than I expected. I waited for almost three hours…. FGD 6-Participant

Another participant complained about lengthy approval processes that prompted cash payments in emergencies, saying,

…where it is most horrible is at the approval process, … you wait for three hours and then you are like no, they tell you, just wait, just wait! Here, you kill a day. Be ready to kill a day when you come. However, even when you go there and you have an emergency, approval takes almost three to four hours. Therefore, for someone who has an emergency, they will have to pay cash. FGD 6-Participant

A client also commented on being socially well known “having connections” as a precursor to timely access to insurance service, saying,

…if you don’t have connections, they take long to work on you, which becomes a problem. FGD 4-Participant

Very few respondents expressed a satisfactory opinion about their waiting time. This was minimal to the extent that they felt that no time was wasted. One participant said,

I remember, the reception was better. They are too quick. They don’t waste time …. FGD 2-Participant

#### Prompt attention- “prompt care received by a few”

Friendly handling and adequate involvement of clients in service delivery processes contribute to service quality. Psychologically, the client is positively inclined to receive quality health services to the best of his or her expectations. In this study, clients mentioned that some healthcare provider staff behaved in an unfriendly manner. The poor attitude and impoliteness of these health workers was registered. A few providers commented on the exaggerated social status attitude among clients. A client commented about the poor health worker attitude, saying,

The attitude issues! …Therefore, every time I called that same person, he began to get tired. I could feel he is tired. Therefore, they even give you someone else to take care of you, you begin to feel very small. Attitude! FGD 1-Participant

#### Poor attitude toward correction” and “unrealistic expectations”

The impolite handling of clients was also emphasized by some providers. The reported poor attitude among health workers was a result of clients’ misunderstanding of service processes amidst competing activities. In circumstances where providers offered some explanations and guidance on observed service processes, clients exhibited negative attitudes toward understanding the providers’ communication. Some clients expected exclusive “cooperate” handling while at provider facilities, which was reported as central to the norm by the provider. One of the healthcare providers commented,

I realize that most of the insured clients view themselves as cooperate.…they require that kind of service that is high class, that is timely and prompt and probably like everything should be put on hold when they are here. KII-Participant

However, a few participants reported that they were respectfully handled. Some health workers were reported as very friendly and guided clients to different service stations. Waiting clients were approached and asked if they had received the services. One satisfied client was quoted saying,

The attention for me; it was okay, the health workers were friendly like from [deidentified] hospital. l was able to be directed very well and there was a lot of friendliness…. FGD 3-Participant

One participant commented about customer care received,

…they have like ushers, so they are always moving around so when they see that you have been siting for a long time they come and ask; have you been attended to. FGD 3-Participant

#### Soundness of facilities- “clean facilities”

Providing care in a conducive environment guarantees that clients achieve better health outcomes. Clean amenities, adequate space and ventilation provide good aesthetics. These factors contribute to healing, psychological wellbeing and adequate infection prevention and control. Clients reported having accessed care in conducive care environments from all provider facilities. Clients appreciated the high-level maintenance and cleanliness of the facilities. One client said that,

…this place is just sufficient and clean, and everything is in order.” FGD 4-Participant

Another client commented about regular and timely cleanliness, saying,

…these private facilities are putting in some effort to ensure cleanliness of the facilities. For example, one time a patient vomited, and she was trying to clean up…. like every 10 minutes someone is passing through. FGD 6-Participant

### Expectations within the insurer-provider relationship

Service eligibility, periodic assessments and payment mechanisms were key themes under this section. Almost all provider respondents mentioned that clear payment timeliness was stipulated in insurer-provider contracts. However, most insurers inadequately oriented their clients on their insurance policies. On the other hand, while some insurers dedicated some focal point staff to support periodic assessments in a few facilities and lacked them in others, they were selective periodic assessments.

#### Service eligibility issues- “less orientation on the policy”, “unclear benefit packages”

Orienting clients on their expected service items and limits as their benefit package is ideally a function of the insurer during purchase of the policy. The insurer must provide details of services the client shall expect and in what limits while at the provider facility. Any deviations result in service misalignment and thus unmet expectations. In this study, providers reported that they suffered the burden of explaining to clients their service package items and limits, a function of insurers. Some insurers only provided generic service information that was less helpful to the client at the point of seeking care, thus exaggerating the client’s unrealistic demands.

There is someone who told me that the insurance had told them that they can see any private doctor they want. They can take as many drugs as they want. In addition, they can treat their family. Therefore, for them, they thought they can come and take any drug of which insurance limits some drugs…. Therefore, it also goes back to the kind of orientation they were given when they were signing them up. Some of them are told unrealistic things. KII-Respondent

#### Periodic assessments- “selective periodic assessment”

Conducting periodic monitoring of service delivery systems informs compliance with or deviation from the set service delivery processes. Feedback informs remedial actions. Such may include improvement, payment of fines, suspension of services or termination of contracted services. In this case, the study reveals that performance assessments were selectively performed. Deployment of focal point persons at provider facilities was only performed in a few facilities. Where insurer focal persons were absent, clients were denied services since they could not be assisted in responding to their inquiries. In such scenarios, clients perceived that the insurer had sold them unrealistic insurance plans.

…we also went further to put in some places our representatives to see how the whole process is going, may be on standby to see how you treat customers with insurance something of the sort [periodic assessment]. KII-Respondent

…Therefore, if they[clients] are entitled to that benefit and they bounce them, then it makes me(insurer) feel like I made an empty promise to them…So the client looks like you (Insurer) sold to them something that was not realistic. KII-Respondent

#### Payment mechanisms- “delayed facility payments”

Provider payments contribute to a revenue base whose resources are used to maintain and improve responsive services. Clients can be assured of continued access to improved quality services if insurers make payments within agreed timelines. Any deviations result in compromised service quality.

Clients earlier mentioned that insurers delayed making payments to providers. Similarly, insurers expressed disappointment that most providers misunderstood how the health insurance model worked. For instance, some provider staff, especially consulting doctors, prescribed medicines of higher quality that were outside those listed in client service packages. In their opinion, the medicines they prescribed had better treatment outcomes than those that were indicated in client benefit packages. As a result, delays from making such inquiries and negotiating between such opinions showed knowledge gaps on how to effect insurance policies. Partly, this became ground for preference to cash clients, thus negating the principle of equitable service provision. The strong inclination of Uganda’s economy to cash transactions was also mentioned as a key influencer of this provider’s behavior.

Medical providers out there are improving their services but obviously they seem not to understand the medical insurance because Uganda being a cash economy those things of serving a client and you wait for payments do not make a lot of sense for them businesswise…. KII-Respondent

The Insurer’s inability to provide feedback to providers was also of concern. For example, until service suspension threats were made by the providers, there was less effort by the insurer to attend to the critical issues that had been raised to the extent of temporarily halting services.

…it took like two years. Then, I wrote, Dr, we are going to suspend the service… that’s when they responded. That’s when they remembered giving me contact with the new Dr. to resolve the issue. KII-Respondent

## Discussion

This study assessed the responsiveness of health care services among actors: the client, insurer, and provider under private health insurance in Kampala. Key findings showed that although most facilities met the expectations of the clients by providing a conducive care environment, clients experienced long waiting times and received inadequate feedback about the services, while a few received prompt care. Under unknown service items and limits of client service packages, clients ended up receiving low-quality medicines. Generally, most of the expectations of these actors were not met.

Instances where clients waited for long hours due to delayed provider payments by the insurer were unpleasing. Making inquiries about the cause of delayed access to services and approval processes influenced the waiting time. During this process, the providers’ behavior shifted, and preference shifted to attending to cash paying clients. This finding is consistent with studies in both Nigeria [22] and Ghana [35], where providers who were contracted to offer health care services to the insured clients under the National Health Insurance Scheme suffered from delayed reimbursement of claims [36]. Justification for this behavior from providers, similar to reports in the study in Ghana, was that providers sought alternative sources of funding to cater to their operational costs, such as maintenance of facilities, purchase of drug consumables and salary payments [35]. Clients considered such repercussions as not having focused on their needs as expected [37], yet they formed the basis for determining the good performance of a health service delivery system [3].

Additionally, congestion at the provider facilities also contributed to clients waiting longer than they expected. Such occurrences contributed to clients’ withdrawal from accessing services under a health insurance arrangement to opt for cash payment alternatives. This negatively affects stakeholders who would find it difficult to renegotiate client willingness to participate in similar health insurance interventions. Such difficult negotiations may frustrate achieving Uganda’s national targets of the Universal (National) health Insurance scheme as indicated in Uganda’s vision 2040 [38].

Addressing congestion in provider facilities positively contributes to the creation of conducive care environments. As an indicator of a good service delivery performance system, it contributes to clients’ positive health outcomes [39, 40]. It may be unlikely that a very clean environment is maintained in congested circumstances. Ensuring sufficient ventilation and keeping toilets and other amenities sound as preventive measures of infection spread and control is critical [10]. Therefore, when feedback from well-conducted periodic assessments is properly relayed, it informs providers in making decisions that address space adequacy and availability for proportionate client volumes [41].

Except for a few, the inadequate prompt care received by insured clients at the provider facilities was inconsistent with what is needed of the health care workforce according to the Uganda Client Charter [42]. While proper handling is a client’s right, some healthcare staff instead expressed a bad attitude as reported by clients. In a Vietnamese study that assessed the quality of care based on the providers’ perspective and opinion, client attitudes were found to be a major challenge to service delivery. Clients often did not follow the laid-out procedure of accessing procedural service points [43]. Their influence on providers’ behavior is inevitable. Where clients accessed service points without clear prescription, it implied that such clients made unrealistic demands just as expressed in this study.

The clients’ unawareness of service items and limits within their insurance policies also contributed to their receiving poor quality services, including medicines. The involvement of clients in understanding their insurance policy is very helpful in averting such effects [37]. Where healthcare providers deployed service guides, they supported clients in navigating service delivery processes that were much appreciated by the clients. The friendliness in handling clients as they guided them informed the clients’ description as staff being very professional.

Nevertheless, good communication between health care service providers and clients plays a key role in achieving expected outcomes [44, 45]. Differences in cultural backgrounds and socioeconomic status impact the level of effective communication and, subsequently, the expectations borne by either the provider or the client [45, 46]. A poor learning attitude, language barrier, heavy workload and unconducive work environments are among factors that also affect effective communication [47, 48]. Whereas findings from a study in Kenya showed that poor communication caused delays in payments that affected facility-based operations such as salary payments and the purchase of replenishing commodities [49], the same contributed to the unfriendly handling of clients in this study.

## Limitations of the study

We adjusted the eligibility of participants of insured clients who had accessed health services from the initial four (4) months to twelve (12) months due to the coronavirus pandemic interruptions. Therefore, participants might have had challenges in recalling their exact experiences.

Insurer-provider contracts were not disclosed to allow the establishment of exact and additional relationship expectations. The study team relied on their self-reported submissions. For instance, if the payment arrangement was for deductibles line-item budgets, this is known to compromise the quality of services. The claim about low-quality medicines that were issued to clients would then be verified.

## Further research

This study only explored the expectations of health insurance actors under private arrangements. Further research may investigate the expectations of actors from the public domain.

## Policy recommendations

In this study, we find unprofessional practices by insurers, such as delays in payments, overpromising and underdelivering on health insurance policies. The Insurance Regulation Authority may consider making regular monitoring in these key areas among its members.

We also find some negligence among clients in sharing feedback on consumed services. We recommend that IRAs sensitize them to consumer rights and their responsibilities.

Lastly, this suboptimal responsiveness of healthcare services under private health Insurance in Kampala across actors was a result of gaps in (1) timely reimbursement, (2) periodic assessments, (3) benefit package education, (4) coordination, (5) accountability and (6) feedback monitoring.

The NHIS planners may review and ensure that these responsive enabling domains in the current plan are clearly outlined and should be explicitly tagged to the program performance indicators for assured service responsiveness.

## Conclusions

Overall, the expectations of health care services in Kampala under health insurance were not met, thus resulting in low service responsiveness. Stakeholders need to consider holistic health service actor-specific expectations and develop and implement disciplined transformation processes that enable improvement. Redesigning and digitalizing permanent and efficient feedback channels may complement such improvement processes. Notably, increased competition among private health insurers may play a critical role in realizing intended improvements in service responsiveness if they are well mobilized. Similarly, with the anticipated establishment of the National Health Insurance Act, stakeholders may need to pay critical attention to establishing supportive infrastructure and systems. These will enable deliberate actor-led service delivery while focusing on service responsiveness.

## Data Availability

These may be provided upon request via email.

## List of abbreviations

HMOs: Health Management Organizations
HRM: Human Resource Manager
IRA: Insurance Regulatory Authority
LTD: Limited
NHIS: National Health Insurance Scheme
OOP: Out of pocket
PFP: Private for Profit
WHO: World Health Organization
